# Melatonin as Immune Potentiator for Enhancing Subunit Vaccine Efficacy against Bovine Viral Diarrhea Virus

**DOI:** 10.3390/vaccines9091039

**Published:** 2021-09-18

**Authors:** Yi-Xuan Wang, Guang-Hui Yang, Lin-Lin Zhang, Jing Wang, Jiu-Feng Wang

**Affiliations:** College of Veterinary Medicine, China Agricultural University, Beijing 100193, China; wangyixuan20201018@163.com (Y.-X.W.); ygh564701721@163.com (G.-H.Y.); zhanglinlin0902@163.com (L.-L.Z.); wangjing100193@163.com (J.W.)

**Keywords:** BVDV, melatonin, the NF-κB inflammation pathway, T-cell immunity

## Abstract

Bovine viral diarrhea virus (BVDV) is a pathogen associated with substantial economic losses in the dairy cattle industry. Currently, there are no effective vaccines against BVDV. Melatonin (MT) has been shown to have anti-inflammatory and anti-viral properties, and the use of MF59 in vaccines significantly enhances vaccine efficiency. Here, MT and MF59 were added into the E^rns^-LTB vaccine. Subsequently, their inhibitory activity on the NF-κB signaling pathway in Mardin-Darby Bovine Kidney cells and the hippocampus was assessed using western blot and quantitative reverse transcription PCR. The findings revealed that MT in the E^rns^-LTB vaccine decreases the phosphorylation of p65 proteins caused by BVDV infection. In addition, MT decreased the mRNA levels of IL-1β and IL-6 in vitro, but increased the production of IFN-α, IFN-β, Mx1 in vitro, brain-derived neurotrophic factor, cyclic amp response element-binding protein, and the stem cell factor in vivo. Furthermore, treatment with E^rns^-LTB + MF59 + MT stimulated the production of T lymphocytes, alleviated pathological damage, decreased expressions of BVDV antigen, and tight junction proteins in mice. These findings imply that MT has potential for use in the E^rns^-LTB vaccine to inhibit BVDV infection and regulate the immune responses of T-cells by inhibiting the NF-κB signaling pathway.

## 1. Introduction

Bovine viral diarrhea virus (BVDV) is a *Pestivirus* of the family *Flaviviridae* [1,2,3] endemic in wild and domestic animals worldwide [4,5]. Its wide distribution and transmission between various hosts increase the difficulty in controlling its spread. Upon infection on a host, BVDV invades and damages multiple organs and tissues including the brain, kidney, lung, liver, intestine, and spleen by altering the major component of the cells such as the tight junction proteins (TJs) [6,7,8]. Tight junction proteins such as occludin and claudin-1 maintain epithelial barrier functions, thus, their alterations increase the paracellular permeability [9] where BVDV uses them as co-receptors for cell entry [8]. Brain damage is mainly manifested in cattle by neurological symptoms, inflammation, and eventually memory and cognitive impairment [10,11]. This results in a decline in fecundity and milk production in dairy cows, leading to incalculable losses in the dairy industry [12,13].

In cattle, BVDV is prevented through vaccination and eradication of persistently infected cattle before the virus causes irreversible organ damage [14,15]. Modified live vaccines and inactivated vaccines against BVDV-1 and BVDV-2 strains are commercially available in many countries. However, the use of these vaccines is faced with numerous challenges. For example, their cross-protection against different BVDV strains is limited. Moreover, modified live vaccines have potential safety hazards. Due to repeated inoculation and passage, BVDV may appear atavism, resulting in enhanced virulence. On the other hand, inactivated vaccines are expensive to produce [16]. Compared with modified and inactivated vaccines, subunit vaccines are safer and offer effective protective responses against BVDV.

BVDV consists of a single-strand positive-sense RNA of approximately 12.5 kb with a single open reading frame (ORF) and non-coded regions on both ends [17]. The ORF is prone to post-translational modifications such as proteolytic processing. After proteolytic and host processing, the BVDV genome is organized into virion proteins consisting of NH2-Npro-C-E^rns^-E1-E2-p7-NS2-NS3-NS4a-NS4b-NS5a-NS5b-COOH [18]. The E^rns^ protein is a structural protein that is well conserved and plays a key role in immune responses. Therefore, the effectiveness of a vaccine can be enhanced by combining the B subunit of enterotoxigenic *E. coli* heat-labile toxin (LTB) with E^rns^ protein [19].

Inflammation caused by BVDV infection will activate the NF-κB signaling pathway. The NF-κB signaling pathway mediates the expression of genes implicated in the body’s inflammatory and immune responses. However, NF-κB is maintained in an inactive state in the cytoplasm by IκB isoforms. When the cells are stimulated, IκB kinase is activated, leading to the phosphorylation of IκB proteins, which causes IκB to dissociate from the inactive NF-κB trimer complex. This activates the NF-κB signaling pathway, leading to the expression of pro-inflammatory cytokines such as IL-1β, IL-6, and TNF-α [20]. The phosphorylation and translocation of p65 also play a crucial role in the NF-κB signaling cascade [21].

Melatonin (MT), one of the hormones secreted by the pineal gland, plays a crucial role in physiological and pharmacological functions in the anti-inflammatory and immunomodulatory processes inhibiting virus replication [22,23]. In anti-inflammatory responses, MT mainly decreases the level of pro-inflammatory cytokines (such as IL-1β) to a normal level [24,25], inhibiting the activation of the NF-κB signaling pathway [26,27]. Currently, MT is successfully used to treat viral infections, sleep disorders, respiratory diseases, viremia, and paralysis [28]. In addition, it is used as a therapeutic and neuroprotective agent to treat pediatric epilepsy due to its antioxidant properties [29]. In sheep, MT increases the immune responses following vaccination against *Dichelobacter nodosus* [30]. However, the use of MT as an immunopotentiator in subunit vaccines against BVDV has not yet been explored.

MF59 is a safe, high tolerant oil-in-water adjuvant used in licensed vaccines, which elicits the production of neutralizing antibodies and activates T-cell responses, potentiating cross-protective immunity [31,32]. The use of MF59 in subunit vaccines enhances the vaccine efficiency in conferring immunity [33,34]. Therefore, we hypothesized that the addition of MT and the use of MF59 in the E^rns^-LTB subunit vaccine could enhance its anti-inflammatory and immunomodulatory effects, subsequently enhancing the immunoprotective efficacy of the BVDV vaccine. This study aimed to explore the effect of MF59 as an adjuvant in combination with MT on the immunoprotective efficacy of the E^rns^-LTB subunit vaccine. The findings of this study demonstrate that as an immune booster, melatonin could be combined with vaccine adjuvant to improve the immune efficacy of vaccines.

## 2. Materials and Methods

### 2.1. Ethics Statement

All the experimental procedures were reviewed and approved by the Animal Ethics Committee of China Agricultural University (permit number: AW12501202-2-1). All surgeries were performed under anesthesia (sodium pentobarbital), and all efforts were made to minimize animal suffering.

### 2.2. Virus, Plasmids, and Cells

A BVDV strain, BVDV1-NADL (GenBank Accession Number M31182.1), was obtained from the China Veterinary Culture Collection Center (Beijing, China). The *pET32a* vectors were purchased from Takara Biomedical Technology Co. Ltd. (Beijing, China). The recombinant plasmid (pET32a-E^rns^-LTB) and BVDV-free Mardin-Darby Bovine Kidney (MDBK) cells were preserved in our laboratory.

### 2.3. Cell Proliferation Test

The effect of MT on cell proliferation was assessed in vitro using the Cell Counting Kit-8 (CCK-8, Beyotime Biotechnology, Shanghai, China) following the manufacturer’s instructions. MDBK cells were seeded into 96-well plates (100 μL per well) and incubated for 24 h. When the cells were about 80% different confluent, concentrations of MT (0.1, 0.3, and 0.5 mM) were added to the wells and incubated for 36 h or 48 h. After that, the wells were washed repeatedly three times with phosphate-buffered saline (PBS), followed by adding 100 μL medium and 10 μL CCK-8 solution in each well. The wells were maintained for one hour at 37 °C in a 5% CO_2_ atmosphere, and then the absorbance was measured with a microplate reader at 450 nm.

### 2.4. Cell Culture

The MDBK cells were cultured in RPMI-1640 medium (Gibco, NY, USA) supplemented with 10% fetal bovine serum (Gibco, USA) and 1% streptomycin/penicillin (Gibco, USA) at 37 °C in a 5% CO_2_ atmosphere. When the cells were 80% confluent, they were randomly assigned into three groups: (i) control: the cells were treated with serum-free medium; (ii) BVDV: the cells were treated with BVDV (MOI = 5) for 1 h; and (iii) BVDV + MT: the cells were treated with MT (0, 1, 0.3 and 0.5 mM) [34] and immediately treated with BVDV (MOI = 5) for 1 h [35]. All treatments were performed in six-well plates.

### 2.5. Protein Extraction and Western Blotting

The total proteins in the MDBK cells were treated with the serum-free medium. BVDV and BVDV + MT were extracted 36 h post-infection using the BCA Protein Assay Kit (Thermo Fisher Scientific Co. Ltd., Shanghai, China) according to the manufacturer’s guidelines. The extracted proteins were separated by reducing SDS-PAGE electrophoresis and transferred onto a PVDF membrane blocked with 5% nonfat milk in Tris-Tween-buffered saline buffer for 1.5 h. The membranes were then incubated with primary antibodies [IκB, p-IκB, p65, and p-p65 (all 1:1000 from Abmart, Shanghai, China) and E2 (1:1000)] and secondary antibodies [Goat anti-mouse IgG (1:5000, SA00001-1; ProteinTech Group, Rosemont, IL, USA) and Goat anti-rabbit IgG (1:5000, SA00001-2; ProteinTech Group, USA)] conjugated with horseradish peroxidase. The gray values for each band were quantified using ImageJ software and normalized to those of GAPDH (1:5000, 10494-1-AP; ProteinTech Group, USA). E2 antibody was generated in rabbits and produced by our laboratory.

### 2.6. RNA Extraction and RT-qPCR

The RNA was extracted and purified from MDBK cells using the Trizol reagent (TakaRa, Beijing, China) following the manufacturer’s guidelines. The total RNA was reverse transcribed to cDNA using the PrimeScript RT Reagent Kit with gDNA Eraser (TakaRa, Beijing, China). The cDNA products were used as templates for (RT-qPCR) using primers shown in Table 1 [6]. The thermocycling conditions were set as follows: initial denaturation for the 30 s at 95 °C followed by 40 cycles of 15 s denaturation at 95 °C, annealing for 30 s at 62 °C, and extension for 30 s at 72 °C. The mRNA abundance was estimated using the Ct method, where the relative expression levels of mRNA were analyzed using the 2^−ΔΔCt^ method. The Ct values normalized with GAPDH were used to calculate the mRNA-expression levels of IL-6, IL-1β, IFN-α, IFN-β, M × 1.

### 2.7. Animal Design and Sampling

Thirty-two six-week-old SPF (Specific Pathogen Free, SPF) female BALB/c mice were purchased from Beijing Vital River Laboratory Animal Technology Co. Ltd. and maintained in pathogen-free, individually ventilated cage systems (IVCs) under negative pressure in China Agricultural University Laboratory Animal House. There were four mice per cage fed ad libitum regular pellet. The mice were maintained in a hygienically controlled room with a stable temperature (24 ± 1 °C) and on a 12:12-h light:dark cycle. They were randomly assigned into four groups (*n* = 8 per group) and immunized intraperitoneally thrice (i.e., at day 0, 7, and 14) as follows: (1) Control group: immunized with PBS; (2) BVDV group: immunized with PBS; (3) Adjuvant group: immunized 100 μg of recombinant E^rns^-LTB protein vaccine formulated with MF59 adjuvant; and (4) MT group: immunized 100 μg of recombinant E^rns^-LTB protein vaccine formulated with MF59 adjuvant, and immunopotentiator (MT). The MT (solarbio, Beijing, China) was dissolved in dimethyl sulfoxide (DMSO) at a rate of 500 mg/mL, then diluted with saline to a working solution of 30 mg/mL [36]. Each mouse was injected with 300 μL of the vaccine formulations. The immunization schedule and sampling time point are shown in Figure S1.

Except for the control group, all the mice were challenged with 6 × 10^6^ median tissue culture infective dose (TCID_50_) of BVDV NADL strain at day 28. One week later, the mice were anesthetized, and blood samples were aseptically collected from all the mice in each group. The mice were then euthanized by using the method of spinal dislocation, and their lungs, spleens, livers, kidneys, and colons were immediately collected. The organs and tissues were fixed in 4% formaldehyde and later paraffin-embedded for subsequent assays. The hippocampus and colon tissues were washed with ice-cold sterilized saline and frozen in liquid nitrogen at −80 °C awaiting further analysis.

### 2.8. Immunohistochemistry

Spleen tissues were subjected to immunohistochemical analyses to assess the changes in the number of BVDV antigens. The tissues were incubated with 5% normal donkey serum for 30 min, followed by overnight incubation with the anti-BVDV E2 rabbit monoclonal primary antibody (1:1000 dilution, VMRD, Pullman, WA, USA) at 4 °C in a humidified chamber. After three rinses in PBS, the slides were further incubated with avidin-conjugated goat anti-rabbit immunoglobulin G (IgG) (ChemiCon, Temecula, CA, USA) for 30 min. The slides were washed three times in PBS, and color change was detected using Dako REALTM EnVisionTM Detection System. When the tissue turned brown, they were immersed in distilled water to stop the color development. The tissues were then counterstained using hematoxylin (Beyotime, Beijing, China), treated with 1% hydrochloric acid until the tissue turned blue, dehydrated, and finally observed under an Olympus BX41 microscope (Olympus, Tokyo, Japan) at ×40 magnification. Spleen tissues from unchallenged mice served as negative controls. Images were captured on a Canon EOS 550D camera head (Canon, Tokyo, Japan) mounted on the microscope. The BVDV antigens were scored from 0 to 4 based on the number of positive cells per tissue section as previously described [6]. The scores were as follows: 0 = no positive cells, 1 = 1–10 positive cells, 2 = 11–50 positive cells, 3 = 51–100 positive cells, and 4 ≥ 100 positive cells.

### 2.9. Histology

The lung, liver, kidney, and colon tissues were fixed in 4% formalin, maintained at room temperature for 48 h, and then dehydrated using different alcohol concentrations. The dehydrated tissues were embedded in paraffin and cut into sections with a thickness of 3 μm. The sections were dried in the oven at 60 °C for 30 min, mounted on slides, and stained with hematoxylin and eosin (Beyotime, Beijing, China). The histopathological changes in the lung, liver, kidney, and colon were observed under a light microscope (Olympus BX41, Olympus, Tokyo, Japan) at ×40 magnification. The severity of the histopathological changes was scored as previously described: 0 = no microscopic lesions; 1 = extremely mild; mild edema and desquamation of rare epithelial cells; 2 = mild; slight inflammatory cell infiltration around the small blood vessels; 3 = moderate; edema, slight inflammatory cell infiltration around the small blood vessels, and minor tissue structural damage; 4 = severe; edema, significant inflammatory cell infiltration around the small blood vessels, and serious tissue structural damage [6].

### 2.10. Flow Cytometry

The CD3e/CD4/CD8 triple-color flow cytometry was performed to analyze the differences in the number of lymphocytes among the four groups. First, the whole blood obtained from the mice was mixed with an anticoagulant followed by the addition of erythrocyte lysate buffer. The mixtures were gently pipetted until thoroughly mixed, then maintained at room temperature for 15 min, after which they were centrifuged and the supernatant discarded. The debris and red blood cells were then removed by pipetting from the precipitate. After that, the precipitate was washed twice in PBS, 2 mL RPMI-1640 medium added, centrifuged, and 100 μL of 10% mouse serum in PBS added and maintained for 15 min. The antibodies were dyed as in the previous essay [6]. The stained antibody cells were analyzed on a FACSCaliburTM flow cytometer (BD Biosciences, San Jose, CA, USA) equipped with the FlowJo software (TreeStar, Ashland, OR, USA).

### 2.11. RNA Extraction and RT-qPCR

Total RNA was extracted from the hippocampus using the Trizol reagent following the manufacturer’s guidelines. The extracted RNA processing and RT-qPCR reactions were performed as described in Section 2.6. The relative expression levels of mRNA were analyzed using the 2^−ΔΔCt^ method. The Ct values normalized with β-actin were used to calculate the mRNA-expression levels of brain-derived neurotrophic factor (BDNF), cyclic amp response element-binding protein (CREB), and the stem cell factor (SCF). Primers used in the RT-qPCR reactions were previously described [37] and are shown in Table 2.

### 2.12. Protein Extraction and Western Blot

Total proteins were extracted from the hippocampus and colon tissues using the RIPA buffer (Solarbio, Beijing, China) as described in Section 2.5. Rabbit anti-Claudin-1 polyclonal antibody (1:2000, 13050-1-AP; ProteinTech Group, USA), and rabbit anti-occludin polyclonal antibody (1:1000, 27260-1-AP; ProteinTech Group, USA) were used as the primary antibodies. The gray values for each band of the colon proteins were calculated using ImageJ software and normalized to those of GAPDH.

### 2.13. Statistical Analysis

Statistical analyses of the immune indexes of mice were performed using a one-way analysis of variance (ANOVA) in SPSS (Chicago, IL, USA) version 17.0. The metrics were presented as mean ± standard error. The differences among means were separated using Tukey’s honestly significant difference test for post-hoc multiple comparisons. Data were visualized using GraphPad Prism 7 software (GraphPad Software Inc., San Diego, CA, USA). *p* < 0.05 was considered statistically significant.

## 3. Results

### 3.1. Effects of MT on MDBK Cell Proliferation

There were no significant differences in proliferation among MDBK Cells treated with 0.1, 0.3, and 0.5 mM MT concentrations for 36 h and 48 h compared to the control group (Figure S2). Therefore, 0.1, 0.3, 0.5 mM of MT were considered nontoxic in the subsequent assays.

### 3.2. Effects of MT on the NF-κB Signal Pathway of MDBK Cells

**Figure 1:**
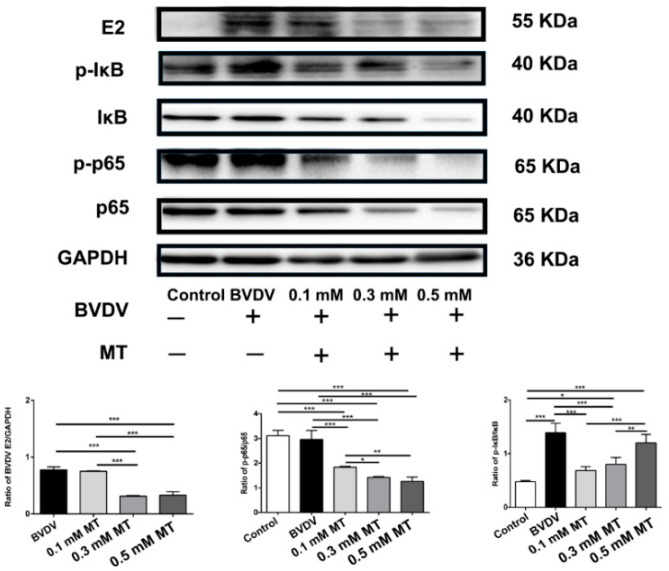
The comparison of the NF-κB related protein expressions in MDBK cells. Relative protein expression levels were based on western blot band intensity; protein levels of p-p65, p-IκB, E2 were normalized, respectively, with p65, IκB, GAPDH. Representative western blot image of NF-κB p-p65, p-IκB, E2, p65, IκB, GAPDH expression levels in the total protein of cells. The values in each column represent the mean ± SEM of three individual experiments detected in triplicate. * *p* < 0.05; ** *p* < 0.01; *** *p* < 0.001.

The expression levels of NF-κB related proteins upon MT treatment were analyzed using western blot assay to assess the effects of MT on the degradation of IκB and transcription of NF-κB p65 in MDBK cells. Our findings revealed that the BVDV-induced inflammation significantly increased p-IκB/IκB levels (*p* < 0.001, Figure 1) compared to the control group. However, the expression of p-IκB/IκB was downregulated when MDBK cells were treated with 0.1 and 0.3 mM concentrations of MT (*p* < 0.001). In addition, the levels of NF-κB p-p65/p65 were significantly reduced upon treatment with 0.1, 0.3, and 0.5 mM MT concentrations compared to the BVDV group at *p* < 0.001. At the same time, BVDV E2 were significantly decreased at 0.3 and 0.5 mM MT concentrations (*p* < 0.001).

### 3.3. Effects of MT on the Levels of Inflammation-Mediating Cytokine in MDBK Cells

**Figure 2:**
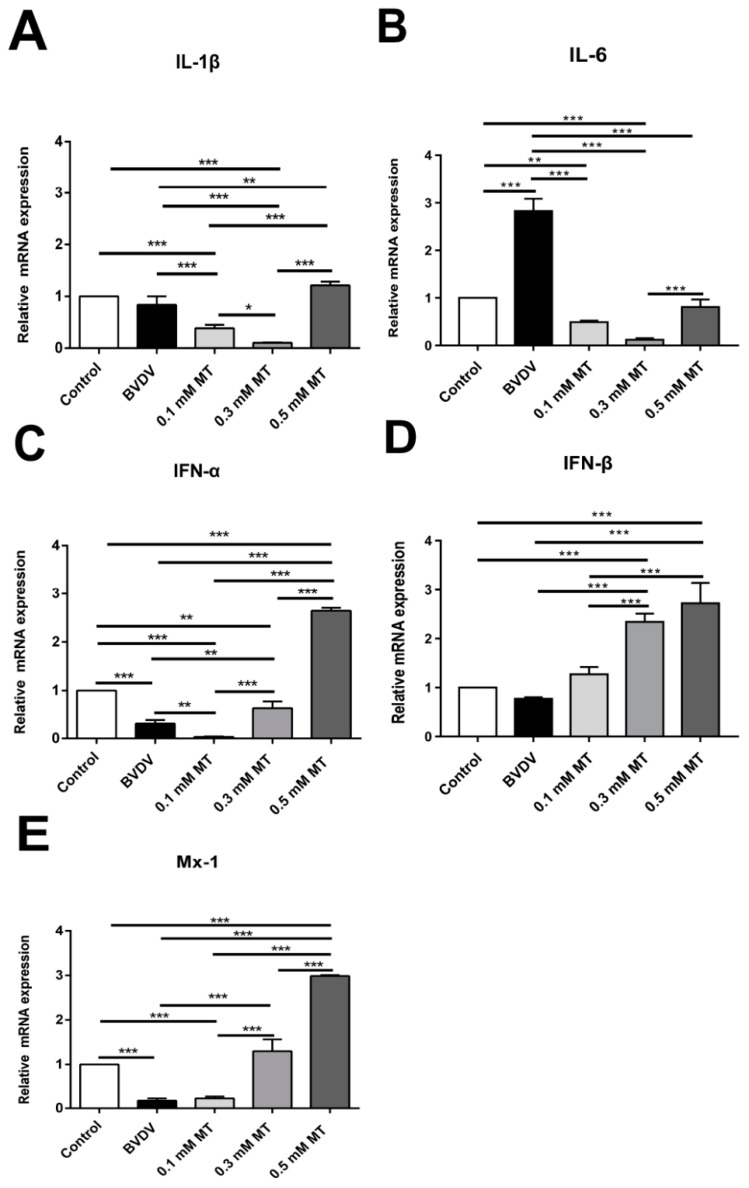
The effect of melatonin on the expressions of cytokines in MDBK cells. MDBK cells were inoculated with BVDV (MOI = 5) in the presence or absence of MT. Total RNA was extracted from cell lysates at 36 h post-infection. The relative expression of (**A**) IL-6 mRNA; (**B**) IL-1β mRNA; (**C**) IFN-α mRNA; (**D**) IFN-β mRNA; (**E**) Mx 1 mRNA was assessed by RT-qPCR. The values in each column represent the mean ± SEM of three individual experiments detected in triplicate. * *p* < 0.05; ** *p* < 0.01; *** *p* < 0.001.

Bovine viral diarrhea virus infection significantly increased the pro-inflammatory cytokine IL-6 compared to the control group (*p* < 0.001; Figure 2B). However, treatment with 0.1 and 0.3 mM concentrations of MT significantly reduced the production of IL-6 (Figure 2B) and IL-1β (Figure 2A) compared to the BVDV group at *p* < 0.001. In addition, MT concentrations of 0.3 and 0.5 mM significantly increased the levels of Mx 1 (Figure 2E), IFN-α (Figure 2C) and IFN-β (Figure 2D) at *p* < 0.001.

Among the three MT concentrations, 0.3 mM had the best inhibitory effect against inflammation. Although the concentration of 0.1 mM decreased the mRNA level of IFN-α, concentrations of 0.3 and 0.5 mM still exerted a positive anti-viral effect, with 0.5 mM having the best effect.

### 3.4. Histopathology and Immunohistochemistry

**Figure 3:**
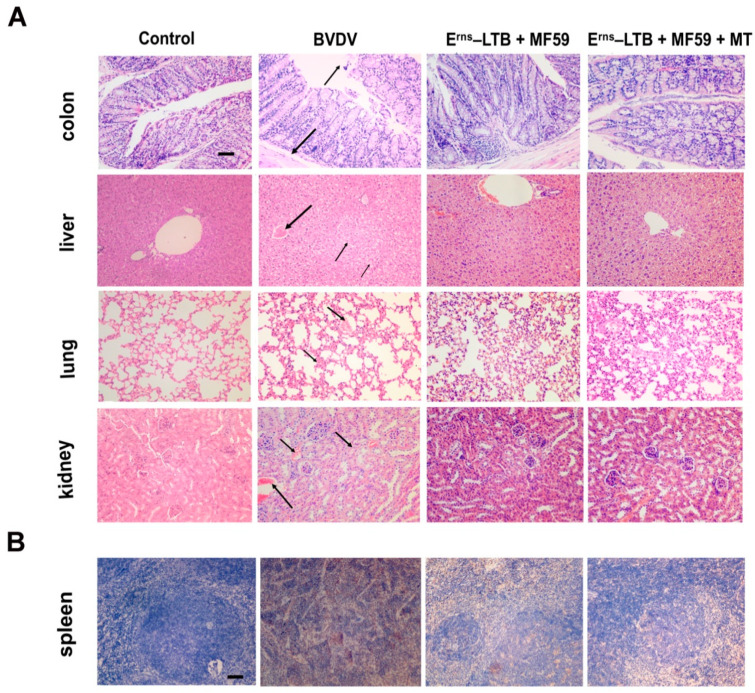
Detection of virus and BVDV particles following BVDV challenge. (**A**) Histopathological changes were observed in the colon, lung, liver, and kidney of the mice from each group. Scale bars, 25 µm. (**B**) The results of BVDV *E2* gene detection in the spleen tissue of different groups after the BVDV challenge.

To evaluate the protective efficiency of the vaccine against BVDV, histopathological examinations were conducted on the lungs, colons, spleens, livers, and kidneys (Figure 3A). It was revealed that there was blood congestion in the central vein of the liver, hepatocellular enlargement, nucleus pyknosis, and liver cord arrangement disorder in the BVDV group. In the adjuvant group, the pathological lesions of the liver were alleviated, while no obvious impairment in the liver was observed in the MT group. In the kidneys, mice in the BVDV group were characterized by blood congestion in the glomerulus, renal interstitial hemorrhage with scattered red blood cells, and an unclear outline of the renal tubular epithelial cells. However, the kidneys of the adjuvant and MT groups showed mild symptoms.

In addition, the capillaries on the alveolar wall were dilated, congested, and the alveolar cavity was filled with a small number of red blood cells in the BVDV group, relative to the vaccine groups. The BVDV group had a characteristic broken intestinal microvillus and incomplete structure in the colon, which was not observed in the adjuvant and MT groups. Furthermore, the submucosa was detached from the mucosa, and the mucosal layer was broken in the colon of the BVDV group, but these changes were minimal in the adjuvant and MT groups.

The number of BVDV antigens was significantly high in the BVDV group than in the MT group. Compared to the adjuvant group, the number of BVDV antigens was lower in the MT group (Figure 3B). The pathological changes and positive BVDV antigens scores in each group are shown in Figure S3.

### 3.5. T Cell Responses Following Immunization

**Figure 4:**
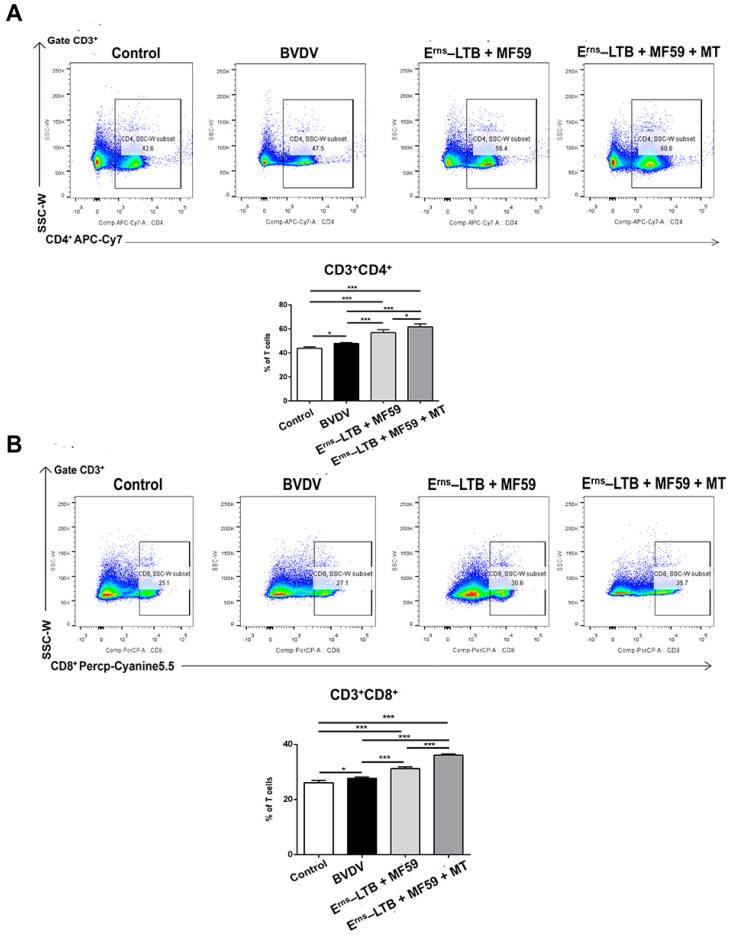
The proportion of peripheral blood lymphocytes CD3^+^CD4^+^ T cells and CD3^+^CD8^+^ T cells. One week after the BVDV challenge, blood was collected aseptically for lymphocyte isolation from mice in each group. (**A**) Flow cytometric analysis of the percentage of CD3^+^ CD4^+^ cells among CD3^+^ T cells. Top: representative flow cytometry dot plot shows the gating strategy for CD4 in peripheral CD3^+^ T cells. Down: The data are the mean percentage of CD3^+^CD4^+^ cells among CD3^+^ T cells. (**B**) Flow cytometric analysis of the percentage of CD3^+^CD8^+^ cells among CD3^+^ T cells. Top: representative flow cytometry dot plot shows the gating strategy for CD8 in peripheral CD3^+^ T cells. Down: The data are the mean percentage of CD3^+^ CD8^+^ cells among CD3^+^ T cells ± SEM of three individual mice from each group detected in triplicate. * *p* < 0.05; *** *p* < 0.001.

To evaluate the effects of MT on cellular and humoral immunity in mice challenged with BVDV, the proportions of CD3^+^CD4^+^ and CD3^+^CD8^+^ cells in peripheral blood lymphocytes were assessed (Figure 4). Notably, all the immunized groups produced significantly more CD3^+^CD4^+^ and CD3^+^CD8^+^ lymphocytes than the BVDV group. Specifically, mice immunized with MT had a higher percentage of CD3^+^CD4^+^ T cells than the other immunized groups where MT was not used (*p* = 0.0138; Figure 4A). Furthermore, the MT group recorded a significantly higher count of CD3^+^CD8^+^ lymphocytes than the MF59 group (*p* < 0.001; Figure 4B). However, mice challenged with BVDV produced more CD3^+^CD4^+^ (*p* = 0.0353) and CD3^+^CD8^+^ (*p* = 0.0118) T cells compared to the control group.

### 3.6. Colon Permeability

**Figure 5:**
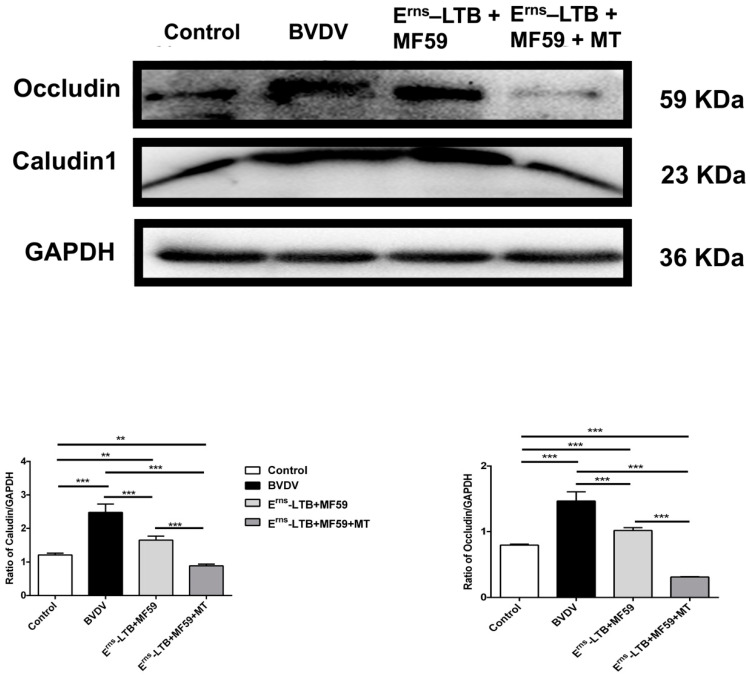
Comparison of the claudin-1 and occludin expression levels in vivo. Relative protein expression levels were based on western blot band intensity; protein levels were normalized with GAPDH. Representative western blot image of claudin-1 and occludin expression levels in mice. The values in each column represent the mean ± SEM of three individual mice from each group detected in triplicate. ** *p* < 0.01; *** *p* < 0.001.

The levels of tight junction proteins (claudin-1 and occludin) were significantly increased in the BVDV group at *p* < 0.001 (Figure 5) compared to the control group. However, in mice immunized with the vaccine containing MT, the levels of claudin-1 and occludin were significantly decreased in the colon compared to the BVDV group at *p* < 0.001 (Figure 5).

### 3.7. MT Inhibits the NF-κB Signaling Pathway in the Hippocampus

**Figure 6:**
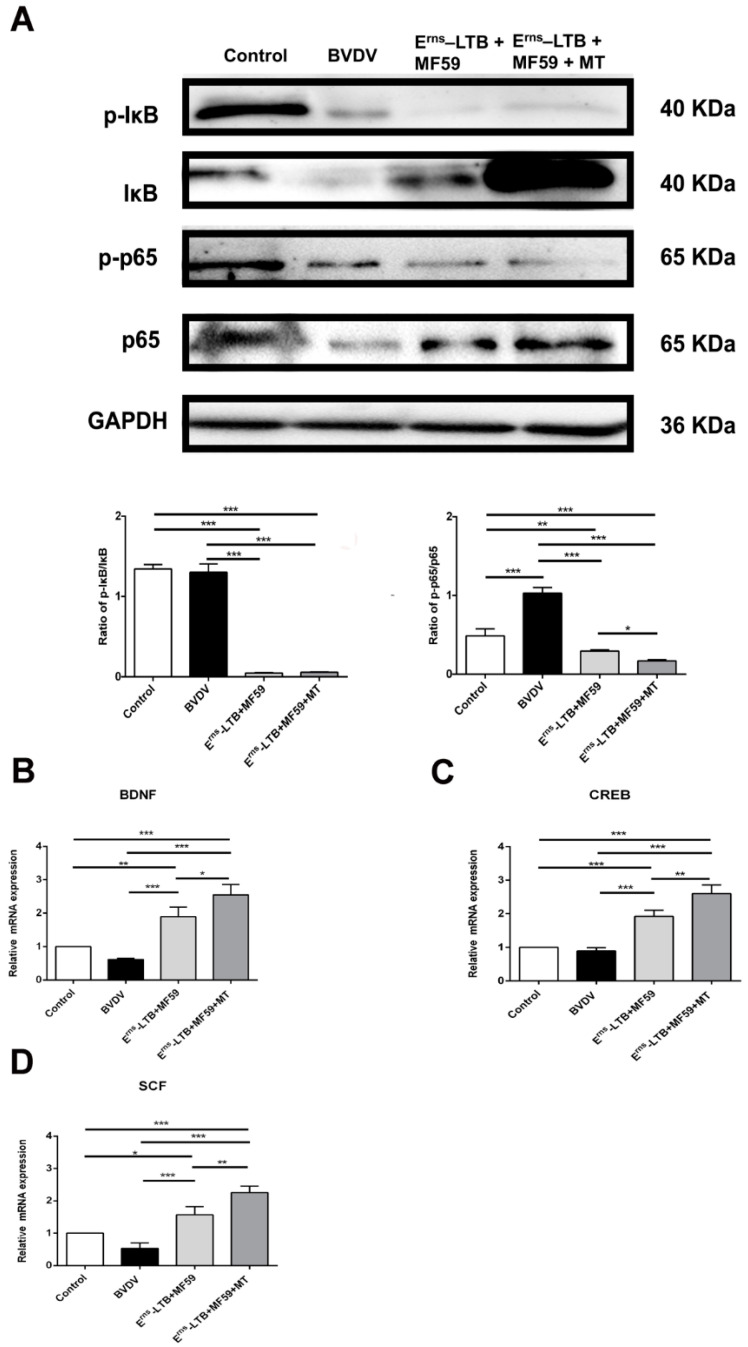
Comparison of the NF-κB related protein expressions, and mRNA levels of memory-related proteins in vivo. (**A**) Relative protein expression levels were based on western blot band intensity; protein levels were normalized with IκB and p65, respectively. Representative western blot image of p-IκB, IκB, NF-κB p-p65, p-65 expression levels in the hippocampus. (**B**) mRNA expression of BDNF; (**C**) mRNA expression of CREB; (**D**) mRNA expression of SCF. The values in each column represent the mean ± SEM of three individual mice from each group detected in triplicate. * *p* < 0.05; ** *p* < 0.01; *** *p* < 0.001.

To confirm the effectiveness of MT in inhibiting inflammation in the hippocampus, the expression levels of IκB, p-IκB, NF-κB p65, p-p65 were analyzed using a western blot assay. In the BVDV group, the ratio of NF-κB p-p65/p65 was significantly increased at *p* < 0.001 compared to the control group (Figure 6A). However, in the MT group, the ratios of p-IκB/IκB and NF-κB p-p65/p65 levels were significantly downregulated (*p* < 0.001) compared to the BVDV group.

### 3.8. MT Repaired the Recognition System in the Hippocampus

The effects of MT treatment in vivo on the expression of memory-related proteins were determined by real-time PCR (Figure 6). In the BVDV group, the memory-related functional proteins (BDNF, CREB, and SCF) were significantly reduced compared to the control group (*p* < 0.001; Figure 6B–D). However, MT treatment with the vaccine significantly increased BDNF, CREB, and SCF mRNA levels compared to the BVDV group (*p* < 0.001; Figure 6).

## 4. Discussion

Given the anti-inflammatory and immunomodulatory properties of MT [38,39], we explored whether MT in vaccines could boost the vaccine’s protective immunity by inhibiting the NF-κB signaling pathway. Supplementing the vaccine with MT elicited the production of T lymphocytes in BALB/c mice and significantly inhibited the NF-κB signaling pathway both in vitro and in vivo.

In vitro, the NF-κB signaling pathway was activated following MDBK cell infection with BVDV, suggesting that BVDV infection induced inflammation. However, immunization with vaccine supplemented with MT before BVDV challenge led to a decline in E2 protein, a structural protein of BVDV, revealing that MT could resist the BVDV infection. In addition, low levels of IκB and p65 in the NF-κB signaling pathway were phosphorylated, indicating that MT has the potential to inhibit the NF-κB signaling pathway, subsequently reducing inflammation in the MDBK cells. In vivo, the p65 protein was phosphorylated into p-p65 in the BVDV group, implying that the NF-κB signaling pathway was activated in the hippocampus. However, in the MT group, p65 and p-Iκb were significantly decreased. Instead, IκB was significantly increased in the MT group, implying that MT has an anti-BVDV effect including reducing inflammation. Thus, MT reduced the activation of inflammation in vitro and in vivo.

In addition, MT inhibited the secretion of pro-inflammatory cytokines IL-6 and IL-1β while promoting the secretion of anti-viral cytokines Mx1, IFN-α, and IFN-β in vitro. Cytokines play an indispensable role in immunity against viral infections by mediating the NF-κB signaling pathway. Therefore, MT could activate innate immunity, subsequently inhibiting BVDV replication and reducing the side effects of inflammation.

To further analyze the influence of MT on the neurological functioning of mice upon BVDV infection, three memory-related functional proteins (BDNF, CREB, and SCF) were identified in the hippocampus. CREB is stimulated by BDNF and serves as a representative marker of synaptic plasticity. SCF is also important in neuronal plasticity since it is related to the memory function of the brain. Infection with BVDV considerably decreases the expression of BDNF, CREB, and SCF, causing memory impairment due to the reduced synaptic plasticity in the hippocampus. However, the negative impacts linked to the exposure to BVDV were neutralized in the MT group, where the MT exerted beneficial effects by protecting the hippocampus from BVDV injury.

It has been confirmed that the BVDV antigens stimulate the proliferation of CD3^+^CD4^+^T cells [40,41]. In this study, the CD3^+^ CD8^+^ T cells in the BVDV group were slightly increased compared to the control group. This implies that CD8^+^ cytotoxic T lymphocyte cells (CTL) positively influenced the immune response to acute BVDV infection. Compared to the adjuvant group, mice in the MT group had more antigen-specific activated CD3^+^CD4^+^ and CD3^+^CD8^+^ T cells. The increased CD3^+^ CD8^+^ T cell responses imply that MT promoted the cross-presentation of MHC (major histocompatibility complex) class I antigens, thereby inducing the CD8^+^ T cell responses. On the other hand, the increased CD4^+^ T cell responses contributed to the secondary expansion and memory of CD8^+^ T lymphocytes [42]. Therefore, the recombinant E^rns^-LTB protein vaccine supplemented with both MF59 adjuvant and MT had a better protective effect than the vaccine supplemented with only MF59.

Furthermore, the TJs (claudin-1 and occludin) levels in the colon were significantly decreased in the MT group. In the early stages of PSaV infection, TJs in the polarized porcine kidney epithelial cells are impaired, leading to increased paracellular permeability. Specifically, occludin binds to PSaV as a co-receptor, while claudin-1 facilitates the entry of PSaV into cells [43]. In this study, treatment with vaccine supplemented with MT inhibited the invasion of BVDV due to the decreased level of receptor proteins occludin and claudin-1. This is consistent with previous findings illustrating that occludin and claudin-1 serve as receptors aimed at helping BVDV invade the epithelial cells [8].

Although this study did not verify the findings on cattle, it lays a solid foundation for further studies. Currently, the combination of anti-viral drugs and vaccines can improve the efficiency of the vaccines in conferring immunity against BVDV. As an immune enhancer in subunit vaccines, MT promotes T cell immunity and plays an immunoregulatory role by inhibiting inflammation. We anticipate that MT can be used as an immunopotentiator in recombinant vaccines because it has a direct inhibitory effect against the virus in vitro and exhibits the ability to stimulate hosts to produce adequate T lymphocytes to resist BVDV infection.

## Figures and Tables

**Table 1:** Sequences of the primers used for RT-qPCR. ^a^ F = forward; R = reverse.

Primers Name	Direction ^a^	Sequence (5′ → 3′)
IL-6	F	GCTGAATCTTCCAAAAATGGAGG
	R	GCTTCAGGATCTGGATCAGTG
IL-1β	F	CCTCGGTTCCATGGGAGATG
	R	AGGCACTGTTCCTCAGCTTC
IFN-α	F	GTGAGGAAATACTTCCACAGACTCACT
	R	TGAGGAAGAGAAGGCTCTCATGA
IFN-β	F	CCTGTGCCTGATTTCATCATGA
	R	GCAAGCTGTAGCTCCTGGAAAG
Mx 1	F	GTACGAGCCGAGTTCTCCAA
	R	ATGTCCACAGCAGGCTCTTC
GAPDH	F	AAAGTGGACATCGTCGCCAT
	R	CCGTTCTCTGCCTTGACTGT

**Table 2:** Sequences of the primers used for RT-qPCR. ^a^ F = forward; R = reverse.

Primers Name	Direction ^a^	Sequence (5′ → 3′)
BDNF	F	GCGCCCATGAAAGAAGTAAA
	R	TCGTCAGACCTCTCGAACCT
CREB	F	CCAGTTGCAAACATCAGTGG
	R	TTGTGGGCATGAAGCAGTAG
SCF	F	CCTTATGAAGAAGACACAAACTTGG
	R	CCATCCCGGCGACATAGTTGAGGG
β-actin	F	GCTCTTTTCCAGCCTTCCTT
	R	GATGTCAACGTCACACTT

## Data Availability

All data generated are contained in the present manuscript.

## Supplementary Materials

The following are available online at https://www.mdpi.com/article/10.3390/vaccines9091039/s1, Figure S1: Immunization and sampling process, Figure S2: The effects of different concentrations of melatonin on cell viability, Figure S3: The scores of pathological changes and number of BVDV antigens after the BVDV challenge.
