# Supplementary material for: Melatonin as Immune Potentiator for Enhancing Subunit Vaccine Efficacy against Bovine Viral Diarrhea Virus
Source: Vaccines (Basel). 2021 Sep 18;9(9):1039. doi: 10.3390/vaccines9091039 (PMC8473004; doi:10.3390/vaccines9091039)

Appendix for "Melatonin as immune potentiator for enhancement of subunit vaccine efficacy against bovine viral diarrhoea virus".

Yi-Xuan Wang, Guang-Hui Yang, Lin-Lin Zhang, Jing Wang, Jiu-Feng Wang*

College of Veterinary Medicine, China Agricultural University, Beijing, China

*Correspondence: jiufeng_wang@hotmail.com; +86 010 6273 1094

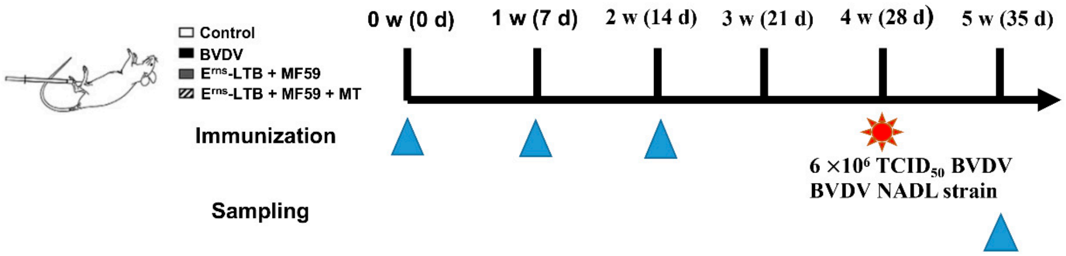

Figure S1. Immunization and sampling process. Thirty-two female BALB/c mice were divided into four groups of n = 8 per group. The different groups were immunized three times intraperitoneally with PBS (control and BVDV groups), with MF59 adjuvanted recombinant E^{rns}-LTB protein or MF59 + MT adjuvanted recombinant E^{rns}-LTB protein. Vaccination took place on days 0, 7 and 14. Four weeks after the primary immunization, the mice were challenged intraperitoneally with 6×10^6 TCID50 BVDV NADL strain. The lung, spleen, liver, kidney, colon of each mouse were collected one week later. The cartoon depicted the method of immunization, and the immunization and sampling schedule were shown in Figure S1.

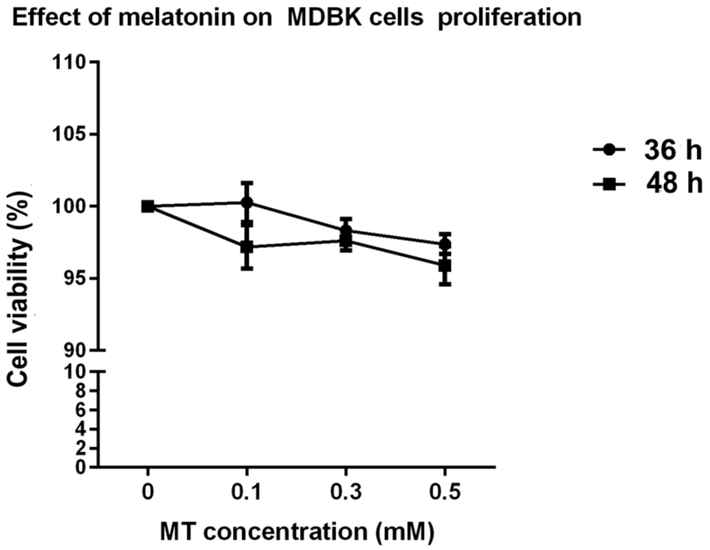

Figure S2. The effects of different concentrations of melatonin on cell viability. MDBK cells were treated with 0.1, 0.3, 0.5 mM melatonin for 36 h and 48 h, respectively. The relative cell viability was evaluated by CCK8 Kit according to the manufacturer's instructions. The values in each column represent the mean ± SEM of three individual experiments detected in triplicate.

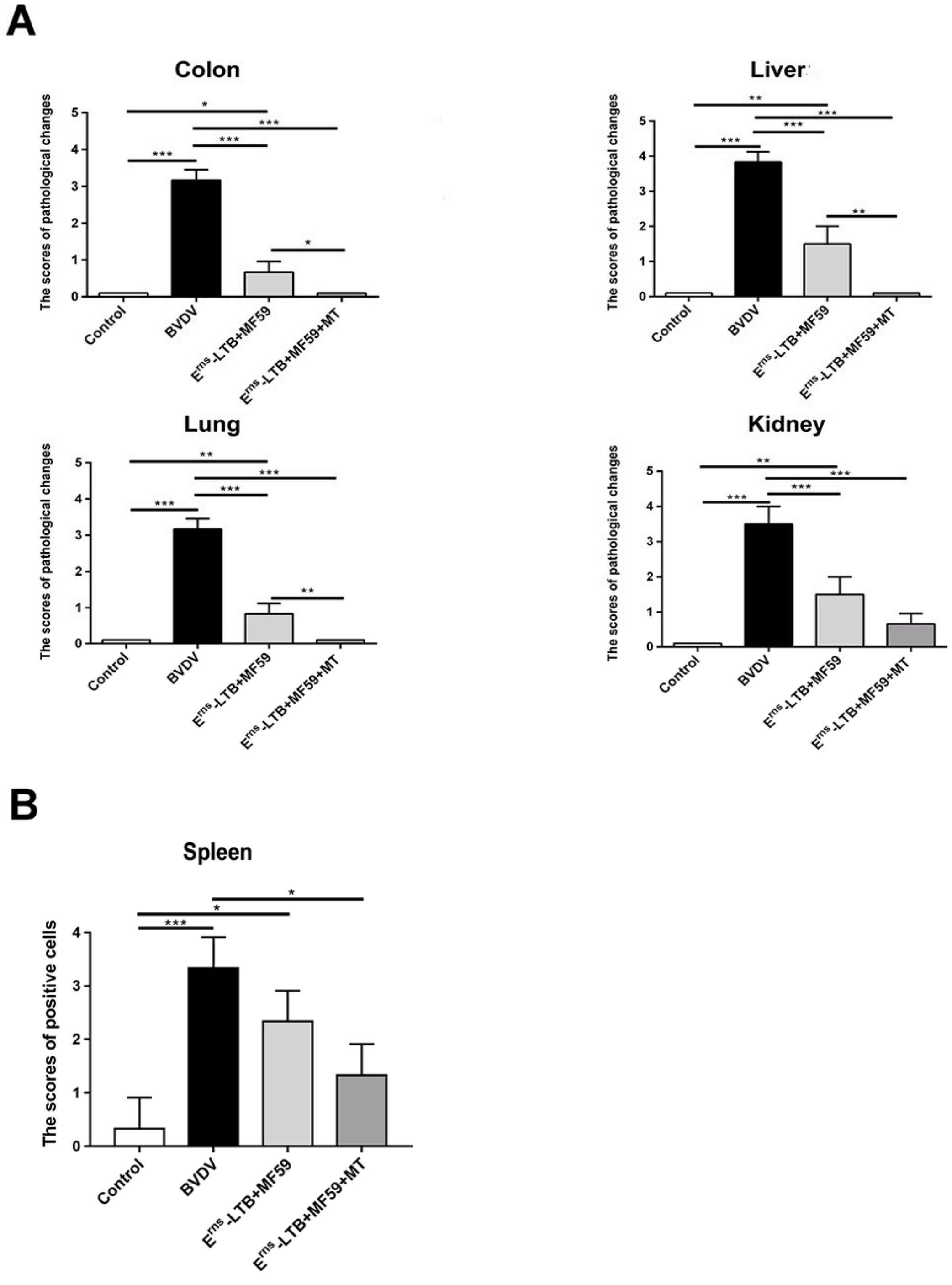

Figure S3. The scores of pathological changes and number of BVDV antigens after the BVDV challenge. (A) On day 7 post-infection, the colon, lung, liver, and kidney of mice in four groups were immediately fixed in 4% formaldehyde. The histopathological changes were evaluated by a veterinary pathologist and scored 0 to 4 in a blinded study. (B) The detection of the BVDV antigen was scored from 0 to 4 according to the number of positive cells per section. * $P < 0.05$; ** $P < 0.01$; *** $P < 0.001$.

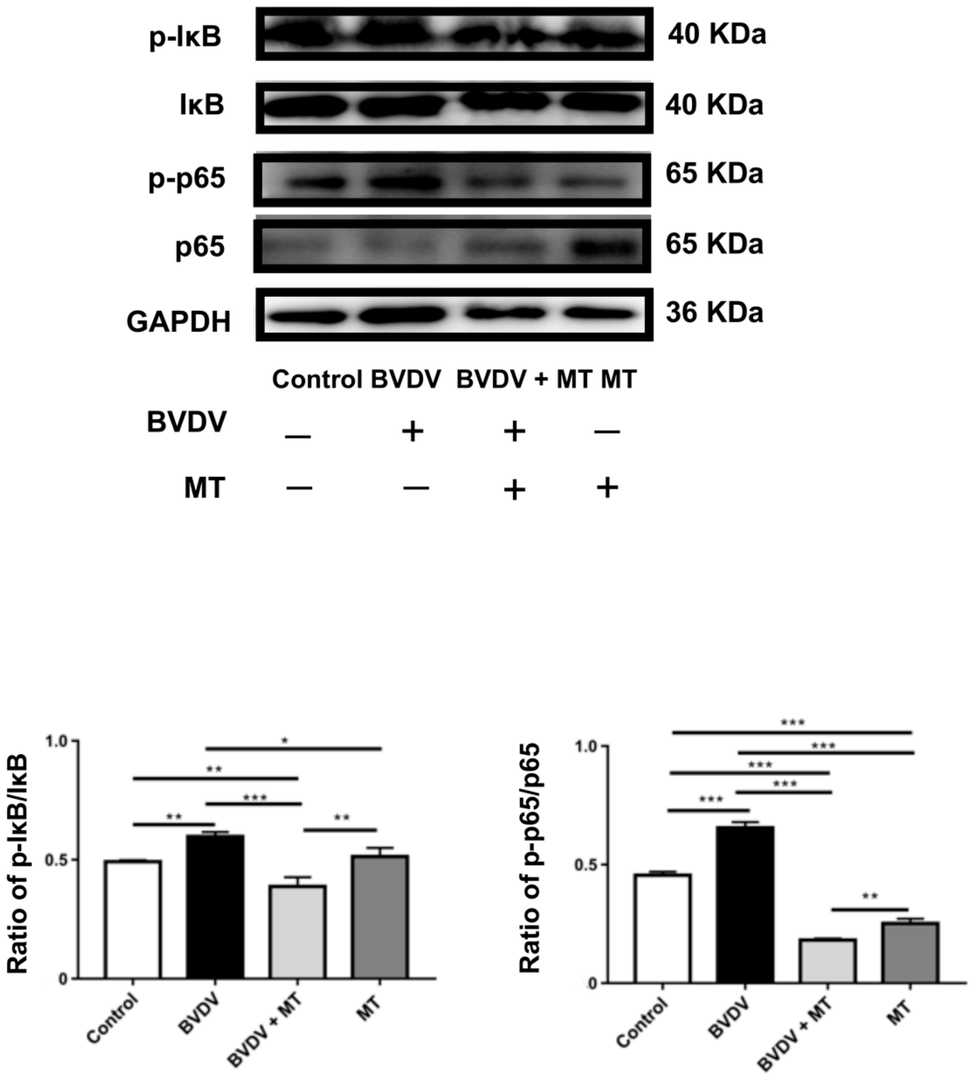

Figure S4. The comparison of the NF-κB related protein expressions in MDBK cells. Relative protein expression levels were based on Western-Blot band intensity; protein levels of p-p65, p-IκB were normalized respectively with p65, IκB. Representative Western-Blot image of NF-κB p-p65, p-IκB, E2, p65, IκB, GAPDH expression levels in the total protein of cells. The values in each column represent the mean ± SEM of three individual experiments detected in triplicate. * $P < 0.05$; ** $P < 0.01$; *** $P < 0.001$.

MogoEdit

CERTIFICATE OF ENGLISH EDITING

This is to certify that the manuscript entitled

Melatonin as immune potentiator for enhancing subunit vaccin e efficacy against bovine viral diarrhoea virus

commissioned to us has been carefully edited by a native English-speaking editor of MogoEdit, and the grammar, spelling, and punctuation have been verified and corrected, except the Figure and table captions. Based on this review, we believe that the language in this paper meets academic journal requirements. Please contact us with any questions.

MOGOEDIT MOGO INTERNET TECHNOLOGY CO., LTD.

Gang Zhang

Dr. Gang Zhang
Founder & CEO of MogoEdit

Date of Issue
August 13, 2021

Disclaimer: The changes in the document may be accepted or rejected by the authors in their sole discretion after our editing. However, MogoEdit is not responsible for revisions made to the document after our edit on **August 13, 2021.**

MogoEdit is a professional English editing company who provides English language editing, translation, and publication support services to individuals and corporate customers worldwide. As a company invested by the affiliate fund of Chinese Academy of Science, MogoEdit is one of the leading language editing service providers in China, whose clients come from more than 1000 universities and research institutes.
MogoEdit Website: http://en.mogoedit.com/
500+ native English editors: http://en.mogoedit.com/editors

Mogo make the world flatter

Mogo Internet Technology Co., LTD.
No. 57, 3rd Keji Road, Xi'an 710075, PR China +86 02988317483 support@mogoedit.com

Uncropped blots
Figure 1
GAPDH-36 KDa

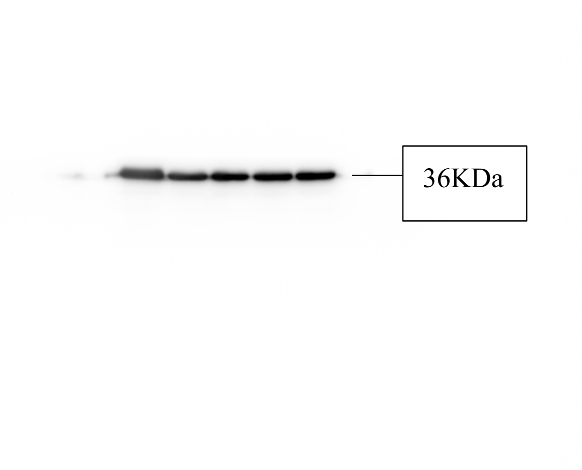

IκB-40 KDa

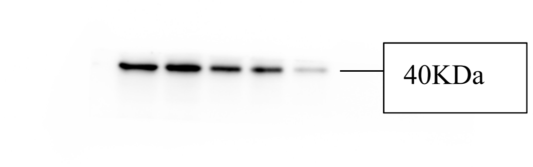

p-IκB-40 KDa

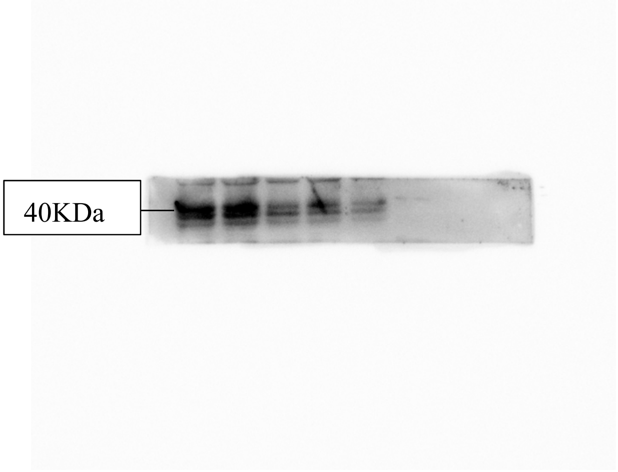

p-p65-65 KDa

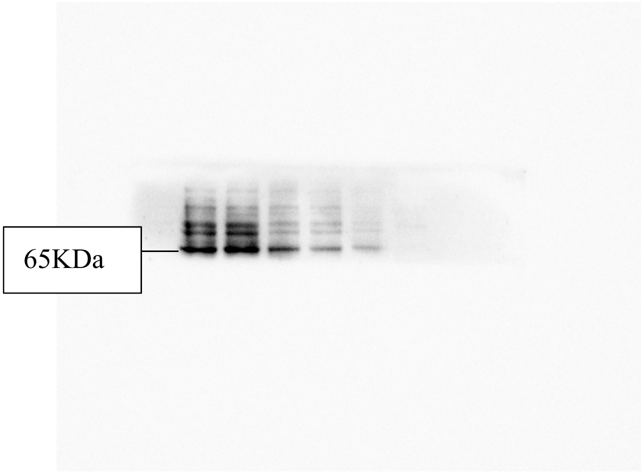

p65-65KDa

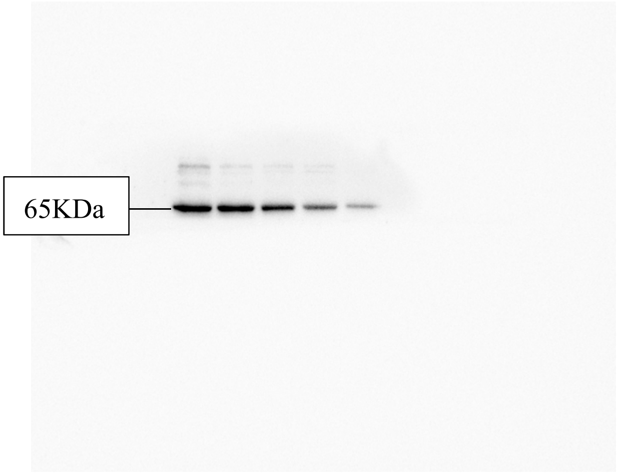

Figure 5

GAPDH-36 KDa

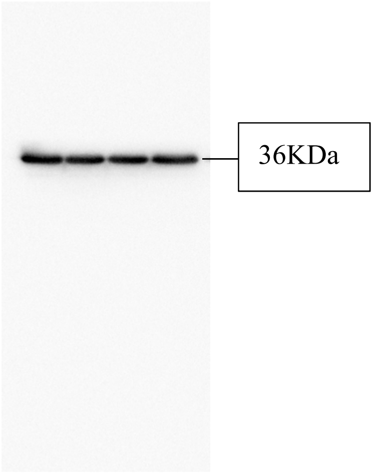

Caludin1-23KDa

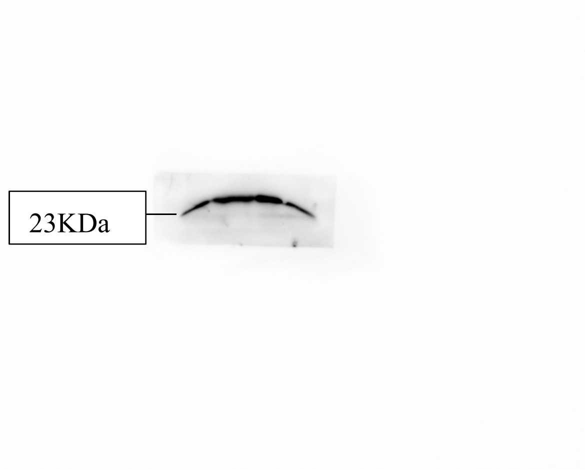

Occludin-59KDa

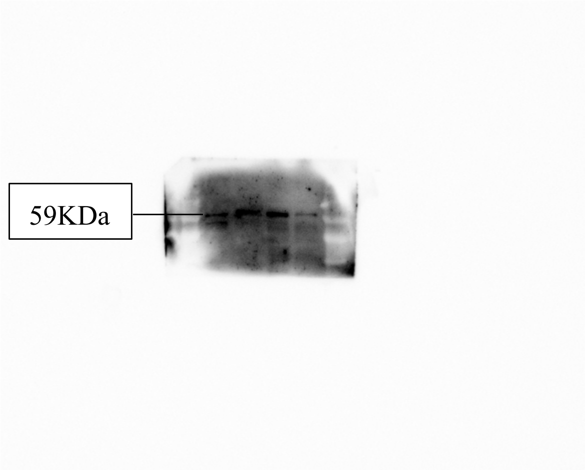

Figure 6
GAPDH-36 KDa

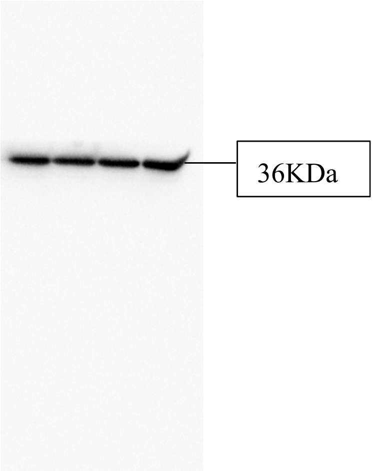

IκB-40KDa

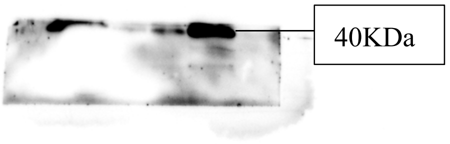

p-IκB-40 KDa

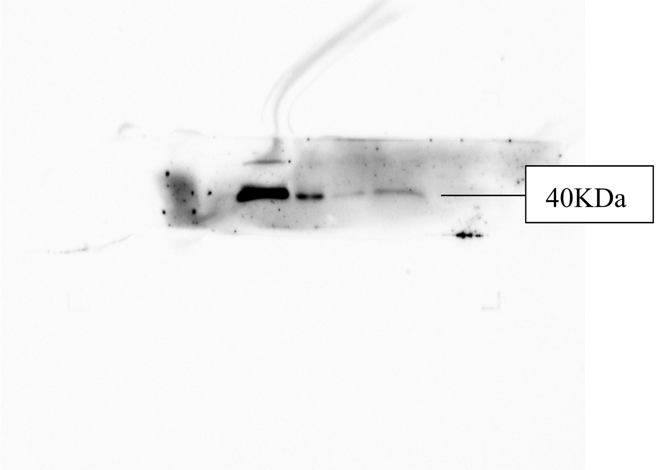

p-p65-65 KDa

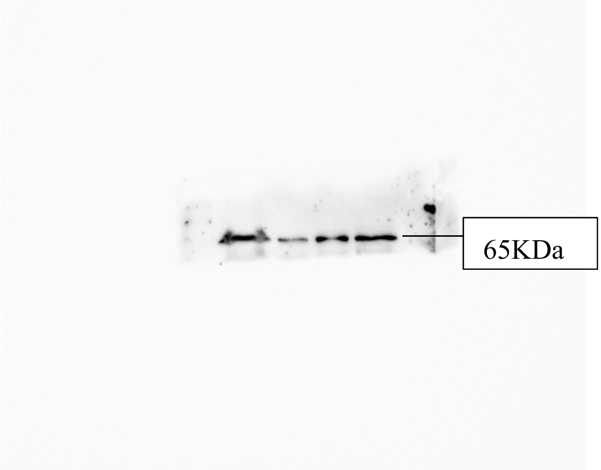

p65-65 KDa

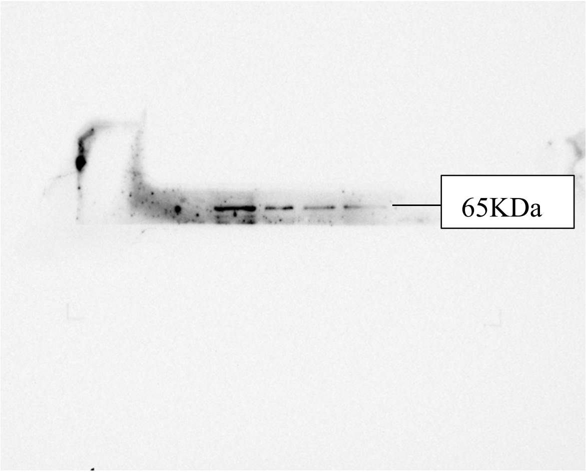

Figure S4

GAPDH-36 KDa

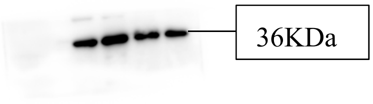

IκB-40 KDa

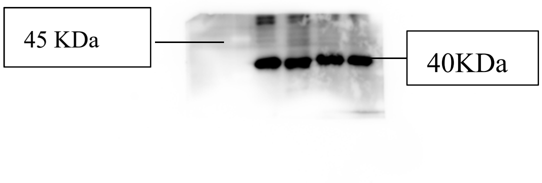

p-IκB-40 KDa

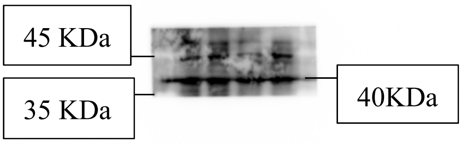

p-p65-65 KDa

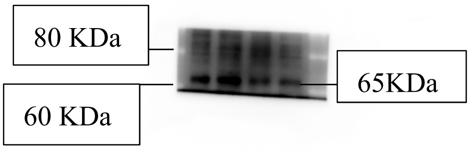

p65-65 KDa

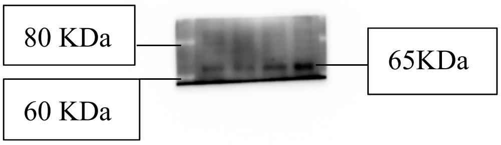

Supplement: Supplementary file 1 [file vaccines-09-01039-s001.zip › vaccines-1361690-supplementary.pdf]
